# A Phylogenetic Analysis of Greek Isolates of *Aspergillus* Species Based on Morphology and Nuclear and Mitochondrial Gene Sequences

**DOI:** 10.1155/2013/260395

**Published:** 2013-05-09

**Authors:** Antonios Krimitzas, Ioanna Pyrri, Vassili N. Kouvelis, Evangelia Kapsanaki-Gotsi, Milton A. Typas

**Affiliations:** ^1^Department of Genetics and Biotechnology, Faculty of Biology, National and Kapodistrian University of Athens, Panepistemiopolis, 15701 Athens, Greece; ^2^Department of Ecology and Systematics, Faculty of Biology, National and Kapodistrian University of Athens, Panepistemiopolis, 15784 Athens, Greece

## Abstract

*Aspergillus* species originating from Greece were examined by morphological and molecular criteria to explore the diversity of this genus. The phylogenetic relationships of these species were determined using sequences from the ITS and IGS region of the nuclear rRNA gene complex, two nuclear genes (**β**-tubulin (*ben*A) and RNA polymerase II second largest subunit (*rpb*2)) and two mitochondrial genes (small rRNA subunit (*rns*) and cytochrome oxidase subunit I (*cox*1)) and, where available, related sequences from databases. The morphological characters of the anamorphs and teleomorphs, and the single gene phylogenetic trees, differentiated and placed the species examined in the well-supported sections of *Aenei*, *Aspergillus*, *Bispori*, *Candidi*, *Circumdati*, *Clavati*, *Cremei*, *Flavi*, *Flavipedes*, *Fumigati*, *Nidulantes*, *Nigri*, *Restricti*, *Terrei*, *Usti*, and *Zonati*, with few uncertainties. The combined use of the three commonly employed nuclear genes (*ben*A, *rpb*2, and ITS), the IGS region, and two less often used mitochondrial gene sequences (*rns* and *cox*1) as a single unit resolved several taxonomic ambiguities. A phylogenetic tree was inferred using Neighbour-Joining, Maximum Parsimony, and Bayesian methods. The strains examined formed seven well-supported clades within the genus *Aspergillus*. Altogether, the concatenated nuclear and mitochondrial sequences offer additional tools for an improved understanding of phylogenetic relationships within this genus.

## 1. Introduction

The genus *Aspergillus* is one of the most common and important genera of microfungi. Several species of the genus proved very useful cell factories for biotechnology purposes since they produce organic acids, extracellular enzymes, nutraceuticals (isoprenoids), and aroma compounds and are capable of fermenting various substrates under extreme cultivation conditions, usually intolerable to bacteria and yeasts [1–4]. Furthermore, the genus contains a number of species that produce toxic metabolites, act as opportunistic pathogens to humans, or decompose a wide variety of substrates [5, 6]. Their high diversity combined with the worldwide distribution necessitates the detailed taxonomic and phylogenetic study of *Aspergillus* species in order to provide more appropriate tools for their accurate and fast identification.

The first most comprehensive taxonomic treatise of the genus was based on morphological criteria and included 150 taxa that were separated in “groups” [7]. These clusters of species (“groups”) were later revised to 18 “sections” in 6 subgenera, in order to receive nomenclatural status [8]. In followup, a list of 182 species valid names was compiled [9], and expanded to ~250 species [3], and it is expected to increase further as new species are discovered and speciation concepts are refined [10]. 

During the last decade, a revision of the genus *Aspergillus* is in progress, as a result of molecular analyses which have offered a better insight into taxonomic and phylogenetic relations [10–13]. The revision is based on polyphasic approaches that include molecular data, as well as morphological, physiological and ecological characteristics. Although no single method worked flawlessly in recognising species, molecular data have largely supported previously inferred relationships that were based on the other characters [3]. Single locus DNA sequence studies were a common practice in fungi in the past, thus, large amounts of information have been accumulated in databases for several parts of the nuclear ribosomal rRNA repeat (in particular the ITS and 28S) [14–16] and genes like the *β*-tubulin [17]. The molecular identification of *Aspergillus* species is currently based on sequences from genes *β*-tubulin, calmodulin, actin, and ITS [5, 12, 13, 18–22]. A phylogenetic study of *Aspergillus* species, based on four single nuclear genes that were concatenated and used as one unit, attempted to resolve the existing taxonomic ambiguities and has resulted in a reevaluation of the genus *Aspergillus* [23, 24].

In other fungal genera, like *Lecanicillium*, *Verticillium,* and *Beauveria*, the use of mitochondrial (mt) genes and mt intergenic regions proved an extremely useful tool to reveal species differences within a genus and even helped to resolve taxonomic ambiguities (e.g., the small rRNA subunit (*rns*), the NADH dehydrogenase subunits 1 (*nad*1) and 3 (*nad*3), the mt intergenic domains *nad*3–*atp*9 and the *atp*6-*rns*) [25–27]. Similarly, other nuclear regions/genes like the RNA polymerase II largest subunit gene (*rpb*1) and the IGS region of the rRNA repeat also proved to be excellent tools for discriminating species within *Metarhizium, Verticillium* and *Aspergillus* [23, 27, 28]. 

In the present study the efficiency of combined nuclear and mitochondrial gene sequence data was evaluated for the identification of species that are distributed in most of *Aspergillus* sections. In detail, the commonly used (ITS1-5.8S-ITS2, RNA polymerase II second largest subunit gene (*rpb*2) and b-tubulin (*ben*A)) and the less often exploited (IGS) nuclear regions were assessed along with two mitochondrial genes, cytochrome oxidase subunit I (*cox*1) and small rRNA subunit (*rns*), for the identification of *Aspergillus* species and the evaluation of their phylogenetic affinities. The objective of this work is to test hypotheses based on morphological criteria and data obtained from a multilocus DNA sequence analysis on the phylogeny within the genus *Aspergillus*.

## 2. Materials and Methods

Several strains of *Aspergillus* have been isolated from the indoor air of a food industry in Greece, as well as from food samples. Additional strains of representative *Aspergillus* spp. were studied from the ATHUM Culture Collection of Fungi in the University of Athens and ex-type cultures were obtained from the Agricultural Research Service ARS (NRRL) at Peoria, USA (kindly provided by Dr. S. Peterson). A total of thirty six strains belonging to thirty-one *Aspergillus* species were studied (Table 1). All strains are maintained in the ATHUM Culture Collection of Fungi.

The strains of *Aspergillus* were three-point inoculated in selective nutrient media and incubated on Czapek Yeast Autolysate Agar (CYA) at 25°C and 37°C, on Malt Extract Agar (MEA) at 25°C, and on G25N at 25°C [29]. The macromorphology of the colonies on each of the media, as well as the micromorphology on MEA, were studied after 7 days of incubation. Microscopic examination was performed by teasing apart the sample in a drop of 70% ethanol on a glass slide, a coverslip was placed on it, and it was observed under a Zeiss microscope in plain light or Differential Interference Contrast. The photographs were taken with an Axiocam digital camera (Zeiss, Germany). When the evaluation of the morphological data was completed, all strains were further examined with molecular markers and the data was used in phylogenetic analyses. Additional taxa were included to represent most of the sections recognized in the genus *Aspergillus*.

Mycelium preparation and fungal DNA extraction procedures used are previously described [30]. PCR primers used in the amplification reactions for the ITS1-5.8S-ITS2 region were 18S-ITS1 and 28S-ITS2 [27] and for the IGS region were CNL12 and CNS1 [31]. For the nuclear genes and specifically for the RNA polymerase II second largest subunit gene (*rpb*2), the primers used were RPB2-6F and RPB2-7R [32] and for the *β*-tubulin gene (*ben*A) the primers were bt2a and bt2b [33]. Primers for the amplification of mt genes were NMS1 and NMS2 for *rns* [34] and cox1F and cox1R for *cox*1 [35]. The PCR amplicons were sequenced using a Thermo Sequenase Primer Cycle Sequencing kit (Amersham Biosciences, Amersham, UK), with a LICOR 4200 IR2 (LI-COR, Lincoln NE, USA) automated sequencer. All fragments were read in both directions and nucleotide sequences were submitted to GenBank database (Table 1).

Maximum parsimony (MP), Neighbour-Joining (NJ), and Bayesian inference (BI) analyses were employed in order to create the phylogenetic trees. MP, and NJ analyses of nucleotide datasets were performed with PAUP* 4.0b10 [36] using 1,000 and 10,000 replicates, respectively, with random addition of taxa and tree-bisection reconnection branch swapping. Reliability of nodes was assessed using 1,000 and 100 bootstrap iterations (for NJ and MP analyses, resp.) and for the NJ analyses the Kimura-2 parameter model was employed. The BI was performed with MrBayes v3.1 [37]. A gamma distribution model of site variation was used, calculated with PAML [38]. For datasets ITS1-5.8S-ITS2, IGS, *ben*A, *rpb*2, *rns*, *cox*1 and the concatenated data, alpha (*α*) was 0.470, 0.630, 0.540, 0.400, 0.348, 0.527, and 0.403, respectively. Random starting trees were used and the burn-in period was 1,000,000 cycles, as this was found to be clearly sufficient for the likelihood and the model parameters to reach equilibrium. After the burn-in, 1,000 trees were sampled every 100 cycles during the sampling period (1,000,000 cycles). Two independent MCMCMC searches were run for each dataset using different random starting points. The hypocrealean fungus *Metarhizium anisopliae* from the Class Sordariomycetes, a close relative to Eurotiomycetes, was used as outgroup in all datasets. When the species *Coccidioides immitis* from *Onygenales*, an order within Eurotiomycetes, was alternatively employed, the tree topologies from all analyses were identical (data not shown). All sequences of *Aspergillus* species and their teleomorphs not amplified in this study were taken from GenBank and are provided with their Accession Numbers and the species names provided by the database. Aligned matrices are available in Treebase, http://purl.org/phylo/treebase/phylows/study/12165. 

## 3. Results

### 3.1. Morphological Analysis

**Figure 1:**
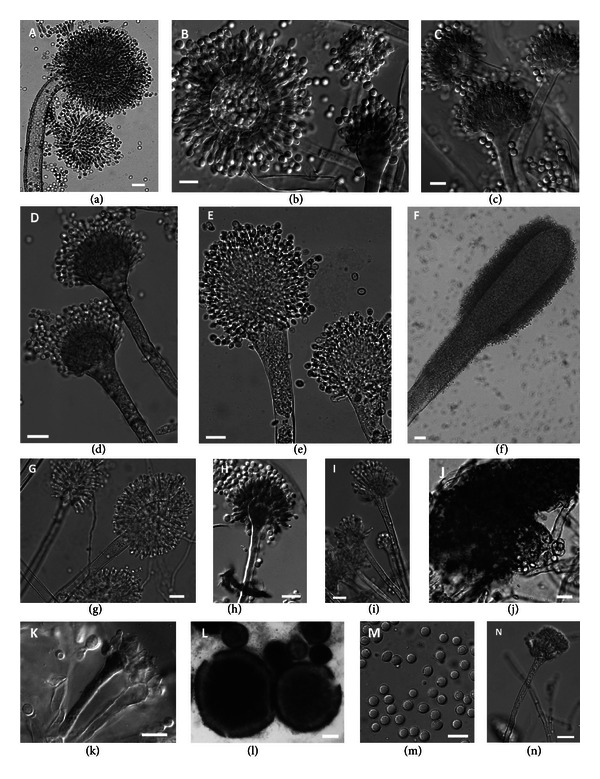
Anamorphic and teleomorphic characters of *Aspergillus* spp. (a) *Aspergillus ochraceus* ATHUM 5014. (b) *Aspergillus flavus* ATHUM 5033. (c) *Aspergillus parasiticus* ATHUM 5037. (d) *Aspergillus fumigatus* ATHUM 5013. (e) *Aspergillus clavatus* ATHUM 5036. (f) *Aspergillus giganteus* NRRL 10. (g) *Aspergillus janus* NRRL 1787. (h) *Aspergillus versicolor* ATHUM 2541. (i) *Aspergillus sydowii* ATHUM 5093. ((j)–(k)) *Eurotium amstelodami* ATHUM 5082. ((l)–(n)) *Neosartorya fischeri* ATHUM 5030. ((a)–(i), (k), (n)) Conidial heads with phialides and conidia. (j) Part of cleistothecium with asci and ascospores. (l) Cleistothecia. (m) Ascospores. Bars = ((a)–(e), (g)–(n)) 10 *μ*m; (f) 20 *μ*m; (l) 50 *μ*m.

The cultural characteristics of the *Aspergillus* spp. have been recorded for all the strains and their evaluation was critical in cases of closely related species. The colony texture, colour and growth rate on the nutrient media, the formation of exudates, soluble pigments and sclerotia, and the thermotolerance were examined. The most important diagnostic characters are the morphological features of the conidial heads, including their growth pattern, size and seriation, dimensions and surface texture of the conidiophore, the shape and size of the metulae, phialides and conidia, and the colour and the wall ornamentation of the phialidospores (Figure 1). The range of variability found for the main morphological characteristics of the strains of *Aspergillus* spp. originating from Greece is presented in Table 2. In addition, the morphology of the teleomorph has been examined in detail when the holomorph was present. There is a great morphological variation in the type of ascomata, from loosely interwoven hyphae to compact sclerotioid structures, as well as in the size, ornamentation, and sculpture of the ascospores.

### 3.2. DNA Analyses

**Figure 2:**
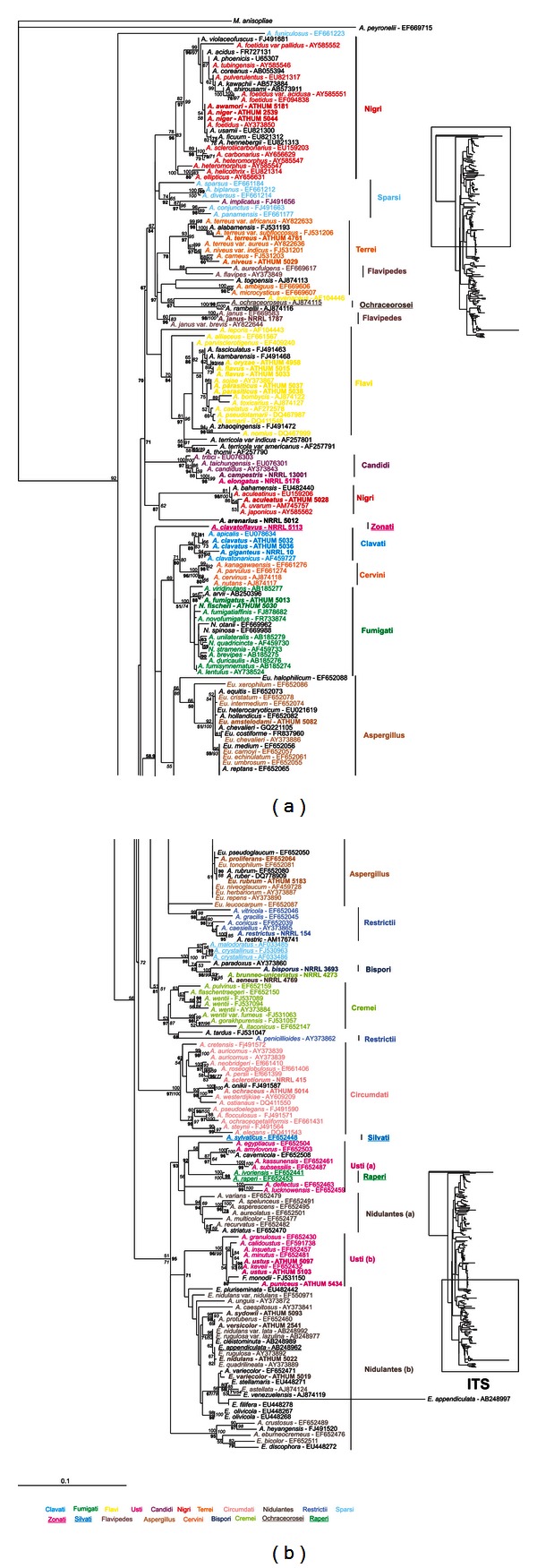
Phylogenetic tree constructed from unambiguously aligned DNA sequences of the ITS domain as produced by NJ. Sequences obtained during this study are presented in bold, whereas those retrieved from GenBank are shown in roman with their Accession Numbers. Clade credibility using NJ calculated from 1,000 replicates (numbers in roman), parsimonial BS support calculated from 100 replicates (numbers in italics) using PAUP, and PPs produced by 1,000,000 generations (numbers in bold) using MrBayes are shown.

**Figure 3:**
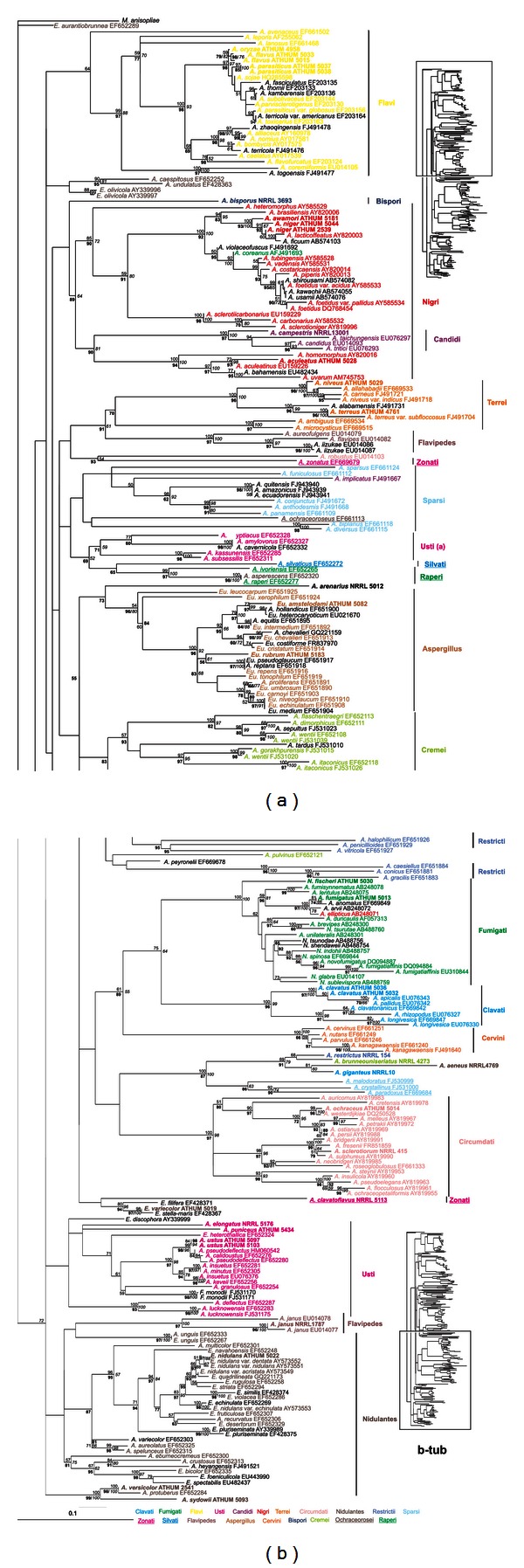
Phylogenetic tree constructed from unambiguously aligned combined DNA sequences of the *ben*A gene as produced by NJ. Sequences obtained during this study are presented in bold, whereas those retrieved from GenBank are shown in roman with their Accession Numbers. Clade credibility using NJ calculated from 1,000 replicates (numbers in roman), parsimonial BS support calculated from 100 replicates (numbers in italics) using PAUP, and PPs produced by 1,000,000 generations (numbers in bold) using MrBayes are shown.

**Figure 4:**
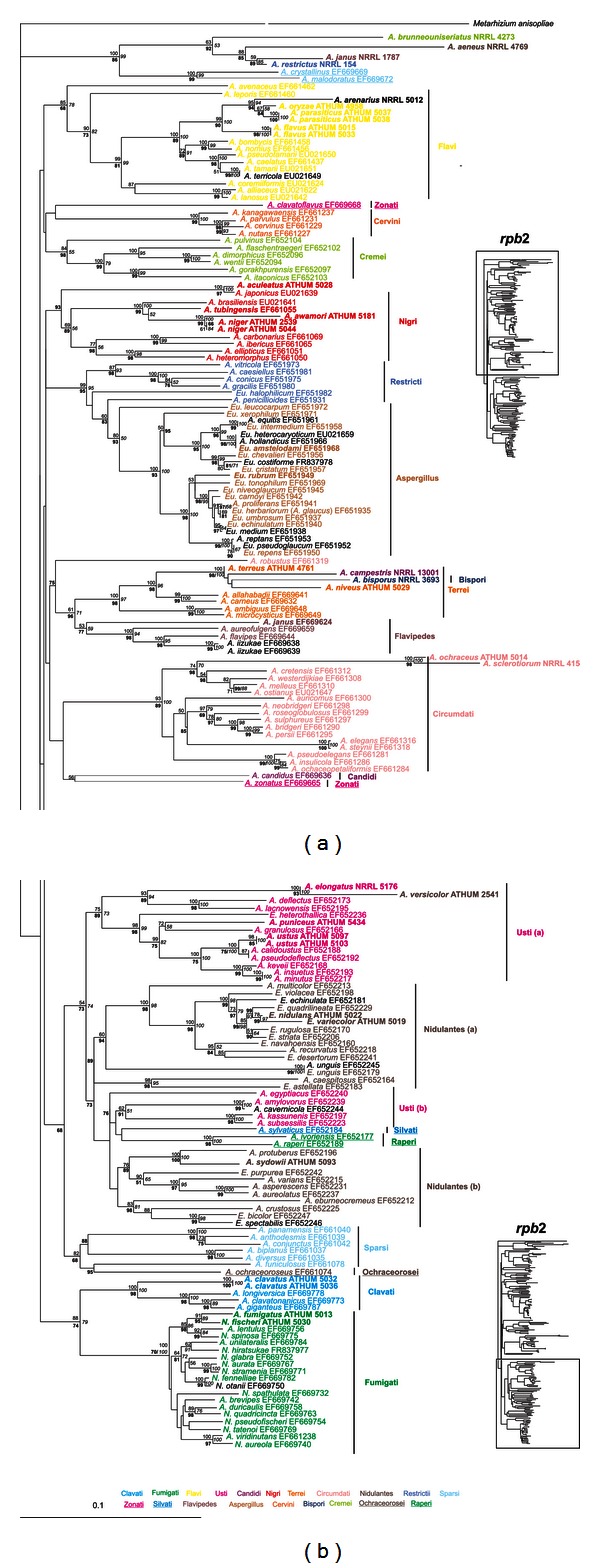
Phylogenetic tree constructed from unambiguously aligned combined DNA sequences of the nuclear *rpb*2 gene as produced by NJ. Sequences obtained during this study are presented in bold, whereas those retrieved from GenBank are shown in roman with their Accession Numbers. Clade credibility using NJ calculated from 1000 replicates (numbers in roman), parsimonial BS support calculated from 100 replicates (numbers in italics) using PAUP, and PPs produced by 1,000,000 generations (numbers in bold) using MrBayes are shown.

**Figure 5:**
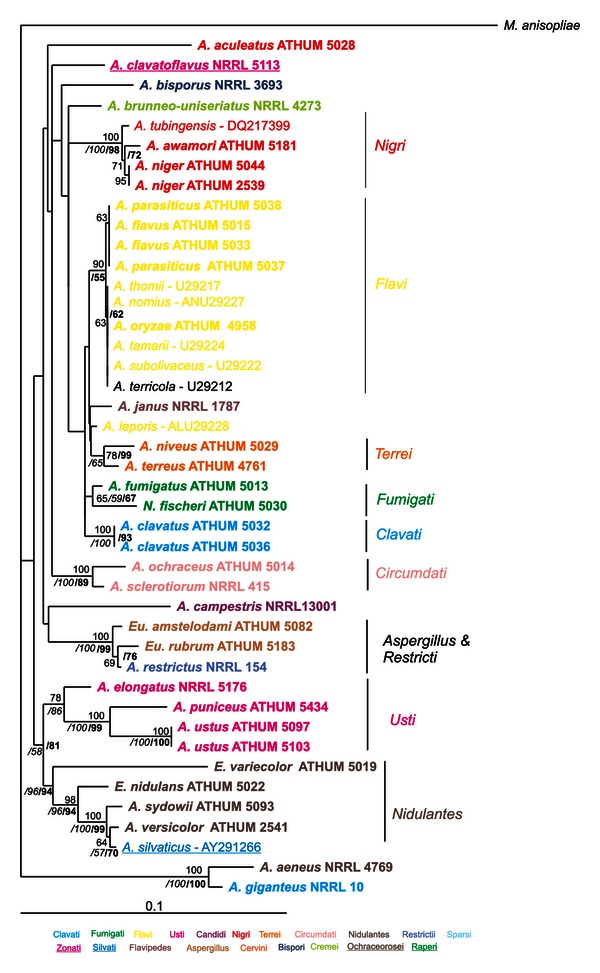
Phylogenetic tree constructed from unambiguously aligned combined DNA sequences of the mitochondrial *rns* gene as produced by NJ. Sequences obtained during this study are presented in bold, whereas those retrieved from GenBank are shown in roman with their Accession Numbers. Clade credibility using NJ calculated from 1,000 replicates (numbers in roman), parsimonial BS support calculated from 100 replicates (numbers in italics) using PAUP, and PPs produced by 1,000,000 generations (numbers in bold) using MrBayes are shown.

**Figure 6:**
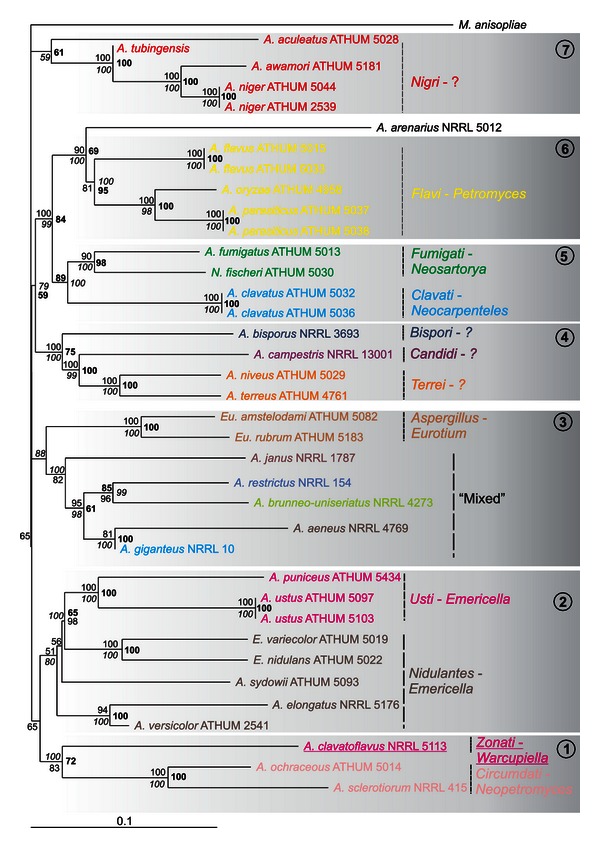
Phylogenetic tree constructed from unambiguously aligned concatenated DNA sequences of the nuclear ITS, IGS, *ben*A, and *rpb*2 genes and the mitochondrial *cox*1 and *rns* genes as produced by NJ. Sequences obtained during this study are presented in bold, whereas those retrieved from GenBank are shown in roman with their Accession Numbers. Clade credibility using NJ calculated from 1,000 replicates (numbers in roman), parsimonial BS support calculated from 100 replicates (numbers in italics) using PAUP, and PPs produced by 1,000,000 generations (numbers in bold) using MrBayes are shown. Solid lines with numbers in circles represent the seven clusters, while the dotted lines show the sections of the species examined and their teleomorphs.

Amplified parts of genes and regions had variable sizes (i.e., in bp, 478–532 for ITS, 650–850 for IGS, 411–587 for *ben*A, 785–811 for *rpb*2, 536–555 for *rns* and 805–847 for *cox*1), reflecting to the apparent genetic variability between different *Aspergillus* species. Amplicons were sequenced in both directions, sequences were combined and compared with all known related sequences in the databanks, and appropriate phylogenetic trees were constructed. Phylogenetic trees deduced from NJ analyses were drawn (Figures 2, 3, 4, 5, and 6), and parsimony and Bayesian methods were used to examine the sensitivity of the resulting trees and tree topologies. Trees remained largely invariant to these manipulations, and topologies were similar for each gene tested independently of the phylogenetic method applied.

The phylogenetic analysis of ITS and *ben*A sequences included 227 and 257 taxa, respectively, belonging to 210 and 235 species of *Aspergillus* and their teleomorphs (i.e., *Emericella*, *Eurotium*, *Fenellia*, *Neocarpenteles*, *Neosartorya*, *Neopetromyces*, *Petromyces,* and *Warcupiella*), in order to determine their phylogenetic positions (Figures 2 and 3). For the datasets of IGS (Supplemental Figure S1 of Supplementary Material available online at http://dx.doi.org/10.1155/2013/260395) and *cox*1 (Supplemental Figure S2) the sequences analysed were significantly fewer than above, since there are no other entries in the databanks apart from those examined in our work (Table 1). Finally, the datasets of *rpb*2 (Figure 4) and *rns* (Figure 5) were enriched with the addition of 147 and 10 more species, respectively, from databanks.

The ITS dataset included 634 characters, 376 of which were parsimony informative, resulting to 10 parsimonious trees (tree length: 2,606, consistency index (CI): 0.360 and retention index (RI): 0.824). Among the 790 polymorphic sites of the *ben*A sequences, 533 were phylogenetically informative. The topology of the NJ tree is the same as one of the 9,980 most parsimonious trees inferred by the PAUP programme (length: 7,986 steps, CI: 0.183, and RI: 0.754). The *rpb*2 and *cox*1 dataset included 1,140 and 908 characters, with 655 and 303 parsimony informative characters, and produced 20 and 1,185 equally parsimonious trees (tree length: 8,927 and 1,185, CI: 0.152 and 0.594, and RI: 0.690 and 0.670), respectively. The IGS dataset included 901 characters, with 735 parsimony informative characters (tree length: 5,829, CI: 0.354, and RI: 0.516). Finally, the *rns* dataset included 614 characters, with 130 parsimony informative characters (tree length: 512, CI: 0.534, and RI: 0.731).

Thirty-six strains studied in this work belong to 31 *Aspergillus* species (see Table 1). ITS sequences from these strains, together with sequences from other species of the genus that were retrieved from the GenBank (altogether around 200), clustered well within their corresponding sections of genus *Aspergillus* (Figure 2). The NJ, MP bootstrap, and Posterior Probabilities (PPs) support of all sections ranged from 51–100%. Members of *Candidi*, *Cervini,* and *Circumdati* clustered with 100% and likewise *Raperi,* and *Restricti* showed higher than 90% support for all three methods. Species of sections *Aspergillus* (*Eurotium xerophilum* and *Eurotium halophilicum* are excluded) and *Flavi* (*A. alliaceus* and *A. leporis* are excluded) presented higher than 90% support for at least two different approaches of bootstrap analyses, whereas sections *Clavati*, *Cremei*, *Fumigati,* and *Sparsi* clustered with excellent support (>90%) for one methodology and at least good (51–89%) for the other two (Figure 2). Species belonging to sections *Nigri, Nidulantes,* and *Usti* were divided into two well discriminated, well supported groups in each case (Figure 2). Subgenus *Nidulantes* based on this dataset seems to be polyphyletic; that is, sections *Nidulantes*, *Raperi*, *Silvati,* and *Usti* form a single cluster, but sections *Bispori*, *Ochraceorosei,* and *Sparsi* are scattered. Finally, strains from different sections, that is, *A. bisporus* NRRL 3693 (*Bispori*), *A. brunneouniseriatus* NRRL 4273 (*Cremei*), *A. aeneus* NRRL 4769 (*Nidulantes*) and *A. paradoxus* AY373860 (*Clavati*) formed a mixed cluster (Figure 2). This cluster grouped as a sister clade to a group consisting of *A. crystallinus* and *A. malodoratus* (see Figure 2) with excellent NJ, MP and PP (100%) support. 

All *ben*A amplicons contained three introns with the exception of *Emericella variecolor* and *A. versicolor* which lack the last intron. As shown in Figure 3, the single tree based on *ben*A nucleotide sequences discriminated clearly species that belong to sections *Candidi*, *Cervini*, *Circumdati*, *Clavati*, *Flavi*, *Fumigati*, *Nigri*, *Raperi* and *Terrei*. Topology support was extremely good for these sections with at least one methodology showing 100% bootstrap value in all cases (except section *Sparsi*: 98, 92, and 62% for NJ, MP bootstrap and PP, resp.). As in the case of the ITS dataset section *Nigri* was divided into two groups with the second group containing five members (i.e., *A. aculeatus, A. aculeatinus, A. bahamensis*, *A. homomorphus,* and *A. uvarum;* Figure 3). Species within sections *Aspergillus*, *Cremei*, *Nidulantes*, *Penicillium*, *Usti,* and *Restricti* although they showed good clustering, they failed to produce high bootstrap support due to the odd discrepancies and must therefore be approached more cautiously.

In accordance with ITS and *ben*A, analyses of the *rpb*2 dataset provided similar or better phylogeny (Figure 4) for species of sections *Aspergillus*, *Clavati*, *Cervini*, *Circumdati*, *Fumigati*, *Penicillium*, *Raperi,* and *Restricti* grouping with excellent bootstrap support (93–100%, irrelevant to the bootstrap methodology applied). Here again, section *Nigri* species were placed into two distinct groups with bootstrap support of 100, 100, 99% and 77, 56, 98% (for the NJ and MP bootstrap and PP of the BI analyses of groups 1 and 2, resp.), but the two groups were sister clades, supported with 69, 56, and 89% NJ and MP bootstrap and PP values, with the exception of *A. japonicus*, which was placed basally to rest of the *Nigri* species. The phylogenies found after the analyses of IGS (Supplemental Figure S1) and *cox*1 (Supplemental Figure S2) did not improve the picture drawn from ITS, *ben*A, and *rpb*2 trees but instead added some ambiguities which could be attributed mainly to the limited number of species examined since this may inhibit the good resolution of the species phylogenetic relationships. Nevertheless, phylogenies from both IGS and *cox*1 can be used for better distinguishing species within the same section (e.g., *A. niger* with *A. awamori*), as also found in a recent paper with other genes by Perrone et al. [39], but are not suitable to discriminate the sections themselves. Finally, the tree produced by the mitochondrial *rns* dataset showed very good clustering of species belonging to sections *Aspergillus, Clavati*, *Circumdati*, *Nigri*, *Nidulantes,* and *Usti* (with the exception of *A. elongatus* which is basal to the other *Usti* species) with excellent bootstrap support (values range from 93 to 100%; Figure 5) and secondarily weaker bootstrap values of 55–90% for species that belong to *Flavi, Terrei,* and *Fumigati* (Figure 5). Thus, a general conclusion from single gene analyses is that subgenera *Aspergillus*, *Candidi*, *Fumigati,* and *Terrei* evidently form well supported clusters, while sections of the subgenera *Circumdati,* and *Nidulantes* are dispersed. 

To achieve full exploitation of the information obtained from nuclear and mitochondrial genes, a tree was constructed based on the combined gene datasets. The concatenated dataset included 4,507 characters, with 2,217 parsimony informative characters and parsimony analysis provided a single tree. The tree length was based on 13,497 steps (CI: 0.40, HI: 0.60, RI: 0.52, RC: 0.21). Analysis of the same dataset with NJ and BI methods produced similar trees with identical topologies wherever there was a strong support (Figure 6). The sections that contained the larger numbers of strains examined, that is, *Flavi*, *Nigri*, *Nidulantes*, along with sections *Aspergillus*, *Circumdati*, *Clavati*, *Fumigati,* and *Terrei*, were well discriminated with better support compared to the results produced by single gene analyses (Figure 6). In addition, the tree produced by the concatenated dataset further supports the polyphyly of subgenus *Circumdati*, as single gene phylogenies have also shown, since sections *Nigri*, *Flavi,* and *Circumdati* form separate clades at the base of the tree. The section *Circumdati*, represented in this study by species *A. ochraceus* and *A. sclerotiorum*, forms a sister clade to section *Zonati*, represented by *A. clavatoflavus.* Both sections can be a sibling clade to section *Nidulantes* based only on the NJ analysis. Both MP and BI analyses show that this relation is not supported and thus sections *Circumdati* and *Zonati* collapse at the base of the tree.

Most importantly, the tree of the concatenated dataset presented a backbone of seven major clades which could be related to the developmental patterns of the teleomorphs (Figure 6). In the subgenus *Circumdati*, clade 1 includes members of section *Circumdati* with connection to *Neopetromyces* [40], a genus with light-coloured multilocular ascostromata. It is distantly related to section *Flavi* (clade 6), with a recently confirmed connection to *Petromyces* [41, 42], characterized by black multilocular ascostromata and section *Nigri* (clade 7) with unknown teleomorphs, even though sclerotia are formed that should be considered as ascomatal primordia [43]. In the subgenus *Nidulantes*, the sections *Nidulantes,* and *Usti* in clade 2 cluster with *Emericella* teleomorphs that produce ascomata surrounded by Hülle cells. Additionally, few species belonging to subgenus *Nidulantes* are in clade 3 which forms a cluster intermixed with subgenus *Aspergillus* sections *Aspergillus* and *Restricti* that contain *Eurotium* teleomorphs with thin layered ascomata loosely suspended in the mycelium. In the subgenus *Fumigati*, in clade 5, the section *Fumigati* is connected to *Neosartorya* with ascomata composed of abundant sterile hyphae and the section *Clavati* is connected to *Neocarpentelles* with unilocular stromatic ascomata. Clade 4 includes taxa in sections *Terrei*, *Candidi,* and *Bispori* without evident teleomorphic connections. 

## 4. Discussion

Fungal phylogeny based solely on morphological criteria or on single genes like the rRNA gene sequences may not always elucidate the taxonomic status of the organisms examined [14]. Also, studies based on a single gene do not always faithfully represent the history of the entire genome containing it and comparisons may give the wrong conclusions about the relationship of a fungus with members of the same species or even the same genus [44]. Certainly, the nuclear rDNA repeat is the most popular region in molecular phylogenetic studies because it is multicopied and contains the highly conserved genes 18S, 5.8S, and 28S, as well as the variable domains of ITS1 and ITS2, and the nontranscribed IGS region. In addition, the 18S and 28S regions often harbour group I introns, inserted at highly conserved positions, contributing further information in regard to this region. These properties have been exploited at length in studies of *Aspergillus* species [11, 15, 45] or other fungal genera [28, 46, 47]. In a similar way, the mtDNA of fungi has lately attracted considerable interest as an alternative or complementary molecule for phylogenetic studies [26, 35, 48], because of its high copy numbers, richness in AT sequences, lack of methylation, universal gene functions, highly conserved regions, as well as variable domains and introns [49]. Currently, the most common mt genes used in fungal phylogenetic studies are *rns* and *cox*1, because the primers designed for these genes can be applied to a wide range of taxonomically different fungi [50, 51]. Our analyses of representative species from the most common sections of *Aspergillus* showed for the first time here that mitochondrial based phylogenies indicate different evolutionary pathways which may provide valuable data for taxonomic purposes when combined with morphological and nuclear based molecular datasets.

It has often been argued that the reconstruction of phylogenies is better achieved by multiple datasets, not only of nuclear but also of mitochondrial origin [52]. In the present study, the ITS and IGS regions from the nu-rRNA gene complex and *rns* and *cox*1 from the mt genomes were chosen to study the phylogenetic relationships of *Aspergillus* species. To overcome possible ambiguities raised by the analyses of these genes, data from the nuclear genes *rpb*2 and *ben*A that are generally accepted as phylogenetically informative were also used [5, 45, 53]. Although *ben*A sequences were excluded from the concatenated data used in the most recent multigene approach, since the presence of two or three introns in the corresponding *ben*A amplicons was attributed to amplification of paralogous genes [23], we have chosen to use this data in our work for three reasons: (a) because 34 out of the 36 species examined contained all three introns, only two species were missing one intron, (b) the identity levels of the 34 amplicon sequences (i.e., containing all three introns) was >84%, and (c) exon identity between all 36 amplicons ranged between 86 and 96%. As shown in results, this choice was justified because the *ben*A based phylogenetic tree provided additional information as, for example, in the case of members of section *Nigri* that were clustered together but clearly differentiated the uniseriate from the biseriate species. Thus, all data from nuclear and mt genes were combined as a single unit in order to blend information from two independent heritage lines, to examine whether this approach provides useful information in resolving phylogenetic ambiguities within the genus *Aspergillus*.

The single gene trees obtained from ITS, IGS, *rpb*2, and *rns* and, mainly, the concatenated datasets, strengthen the fraternisation of *Clavati* to subgenus *Fumigati*, as species of section *Clavati* form a sister group to species within section *Fumigati*, with MP bootstrap and PP support that reached 89 and 100%, respectively, at the combined dataset (Figure 6). This is in full accordance with results obtained from a recent multilocus analysis that was based solely on nuclear genes and the addition of section *Cervini* species in this subgenus [23, 24]. Interestingly enough, our analysis based on the ITS dataset showed that although sufficient bootstrap or posterior probability support was lacking, uniseriate species that belong to subgenera *Fumigati* and *Aspergillus* clustered together, and apart from other species of *Aspergillus* that are biseriate (Figure 2).

Section *Usti* is of particular interest since its elimination and the transfer of species *A. ustus, A. deflectus, A. puniceus, A. granulosus,* and *A. pseudodeflectus* to section *Nidulantes, *and species *A. conjunctus, A. funiculosus, A. silvaticus, A. panamensis* and *A. anthodesmis* to section *Sparsi* was initially proposed [15], only to be rejected a few years later, by exploiting the results of polyphasic approaches in which the sequences of the nuclear *β*-tubulin, calmodulin, actin, and ITS genes were used [5, 18]. These works not only preserved section *Usti* but also added eight more species in it, namely, *A. ustus, A. puniceus, A. granulosus, A. pseudodeflectus, A. calidoustus, A. insuetus, A. kevei,* and *Emericella heterothallica*. Finally, more recent works added nine more *Aspergillus* species in it, seven and two, respectively, bringing the total number to 21 [11, 24]. In full agreement with these, our results suggest that section *Usti* should be retained as an independent section within the subgenus *Nidulantes* with further support, since both the ITS and concatenated data analyses show that species of *Usti, Nidulantes, Silvati,* and *Raperi* comprise a single clade supported (e.g., from ITS dataset) by NJ-MP bootstrap (51% and 71%, resp.), and BI Posterior Probability (95%), and thus, altogether may be considered as one group within subgenus *Nidulantes* (Figures 2 and 6).

Until now, several genes have been proposed for the identification of species within the section of black aspergilli. However, the results obtained are very variable. IGS and *cox*1 sequences were found unsuitable to discriminate species within this section (Supplemental Figures S1 and S2), because they either exhibited too high intraspecies variability or this variability was also extended to inter-species comparisons [3, 20]. Similarly, ITS sequences could distinguish four groups within the *A. niger* species complex but failed to do so for species like *A. carbonarius* and *A. sclerotioniger*, or *A. japonicus*, *A. aculeatus* and *A. uvarum* which had identical ITS sequences [20]. Also, mt cytochrome b (*cob*) gene sequences were informative for many species within the *Nigri* section but they failed to distinguish between *A. niger* and *A. tubingensis* [54]. Thus, the best discriminatory results based on single gene sequences were those obtained from *β*-tubulin and calmodulin phylogenetic analysis which placed 26 taxa within subgenus *Circumdati* section *Nigri,* further dividing them into 5 main clades, grouping all uniseriate species (7) in only one clade (the *A. aculeatus* clade) [13, 45]. In the present work, single gene datasets with many individual sequences (ITS, *ben*A, and *rpb*2) and the concatenated dataset clearly distinguished members of *Nigri* and divided them into two groups: the first containing *A. aculeatus*, *A. aculeatinus, A. bahamensis, A. uvarum—*in all datasets—and *A. homomorphus*, *A. japonicus*—wherever data were available—, and the second composed of all the rest species (see e.g., ITS or *ben*A trees, Figures 2 and 3). It is interesting to note that these two clades correspond to the uniseriate and biseriate conidial head formation, which may be a possible explanation why previous studies that did not take into account this characteristic, failed to cluster them accordingly [13, 20, 45]. An additional explanation for the splitting of section *Nigri* species into two subgroups may be attributed to the range of variability resulting from adaptation of the species to the Mediterranean type ecosystem. This is also evident by the variability found in the morphological characteristics of the Greek *Aspergillus* isolates (Table 2). The biseriate species within section *Nigri* cluster with good bootstrap support (67% bootstrap for NJ and 84% PP) with biseriate species belonging to sections *Flavipedes*, *Terrei*, *Ochraceorosei,* and *Sparsi*, while the uniseriate species of *Nigri* are basal to the above-mentioned biseriate clades of sections *Flavi* with good support (70% PP, Figure 2). Thus, the differentiation of *Nigri* species studied here into uniseriate and biseriate not only helped to group them in combination with molecular markers into two clearly distinct clades, but in addition clustered together biseriate species of all sections. 

Species of the *Flavi* section were grouped well together, with high bootstrap support (>80%), irrelevant to the dataset used. While single gene analysis, like *ben*A, failed to separate *A. flavus* from *A. oryzae,* the concatenated dataset clearly demonstrated its advantages. It could fully discriminate all species examined and could also confirm that taxa within the section, like *A. oryzae*, *A. flavus,* and *A. parasiticus* are phylogenetically distantly related to *A. ochraceus*, as previously proposed [55] and also recently shown with the polyphyly of *A. flavus,* and in extent of section *Flavi* [56]. 

Previous reports showed that sections *Circumdati*, *Flavi* and *Nigri* do not cluster together [23] and this was further confirmed by our phylogenetic analyses of ITS, *ben*A, and *rpb*2 datasets. However, it must be pointed out that this is in contradiction with our morphological observations which demonstrated significant morphological similarities, that is, biseriate conidial heads, large globose vesicles, ascostromata when teleomorph present, and suggest a possible common origin of these sections. The latter is further supported by the analysis of the mt *rns* sequences, even though the bootstrap values do not seem significant enough. Thus, this intriguing controversy between molecular data and morphological observations underlines the necessity to use polyphasic approaches in one hand and increase the number of species examined on the other in order to solve difficult taxonomy problems.

The single tree produced by our concatenated dataset showed that although *A. niveus* and *A. terreus*, which belong to section *Terrei*, are closely related and with excellent support (100% irrelevant of the method applied), they are far apart from *A. janus* (section *Flavipedes*) and *A. arenarius*, both of which were previously placed closely to species of *Terrei*, *Flavipedes,* and *Candidi* [23]. This causes some concern because our dataset places *A. arenarius* as a sister clade to section *Flavi* with good support (bootstrap of 90% for NJ and 100% for MP analyses; 69% Posterior Probabilities for the BI analyses; Figure 6), contrary to suggestions that it is unrelated to any section of *Aspergillus* [24]. Similarly, our concatenated analysis contradicts the proposed merger of sections *Terrei* and *Flavipedes* [24, 57] because *A. janus* is excluded from being a sister clade of section *Terrei* (Figure 6). Since the datasets from the other genetic loci, examined in this work, show a random positioning of *A. janus* and *A. arenarius*, it is obvious that a thorough molecular analysis of the relations between *Terrei* and *Flavipedes* is urgently needed, especially if previous members of *Versicolores,* like *A. janus* and *A. arenarius*, are to be included within these sections. Recently, Jurjevic et al. [58] undertook the study of section *Versicolores* and provided useful phylogenetic data on species of this section based on multilocus DNA phylogeny, but still the inclusion of these species in this section has not been resolved. 

As for the subgenus *Nidulantes*, the tree based on concatenated data showed that it is a compact assemblage of species from sections *Nidulantes* and *Usti*, as expected [23, 24]. Finally, the intermixed cluster of five species from several sections that was not resolved by the analysis of the six genetic loci used in our work is another interesting area of research that will certainly require the introduction of either more genes or more taxa, as it has already been done in other studies facing similar problems [3, 52]. 

In conclusion, our study clearly shows that all molecules used contributed to the resolution of phylogenetic relationships in *Aspergillus*, irrespective to the different ways that the phylogenetic data were processed. It also confirms that single gene based analyses do not solve all ambiguities and do not always represent the evolutionary history of the species. Certainly, *Aspergillus* is a very diverse genus and it is not always easy to determine the phylogenetic relationships between species. Thus, the combination of sequence information from nuclear and mitochondrial genes resolves several circumscriptions and relationships of *Aspergillus* species within different sections and demonstrates the advantages of this multigenic approach. It is expected that the vast amount of information derived from the released complete genomes of *Aspergillus* species will be utilized in future studies by many researchers to select new additional genes from both (nuclear and mitochondrial) evolutionary lines. Such combined data is expected to provide the appropriate levels of resolving power in future phylogenetic studies.

## Supplementary Material

Supplemental Figure 1: Phylogenetic tree constructed from unambiguously aligned DNA sequences of the nuclear IGS region as produced by NJ. Sequences obtained during this study are presented in bold, whereas those retrieved from GenBank are shown in roman with their Accession Numbers. Clade credibility using NJ calculated from 1,000 replicates (numbers in roman), parsimonial BS support calculated from 100 replicates (numbers in italics) using PAUP and PPs produced by 1,000,000 generations (numbers in bold) using MrBayes, are shown.
Supplemental Figure 2: Phylogenetic tree constructed from unambiguously aligned DNA sequences of the mitochondrial cox1 gene as produced by NJ. Sequences obtained during this study are presented in bold, whereas those retrieved from GenBank are shown in roman with their Accession Numbers. Clade credibility using NJ calculated from 1,000 replicates (numbers in roman), parsimonial BS support calculated from 100 replicates (numbers in italics) using PAUP and PPs produced by 1,000,000 generations (numbers in bold) using MrBayes, are shown.

## Figures and Tables

**Table 1:** The strains of *Aspergillus* spp used in this study.

Species	Accession number	Substrate	Origin	GenBank accession number					
				ITS	IGS	*ben*A	*rpb*2	*cox*1	*rns *
*A. aculeatus *	ATHUM 5028^a^	Indoor air	Greece	EU982028	EU982062	EU982087	NT	EU982142	EU982176
*A*. *aeneus* ^T^	NRRL 4769^b^	Forest soil	Somalia	EF652474	EU982060	EF652298	EF652210	EU982140	EU982174
*A*. *arenarius* ^T^	NRRL 5012	Soil	India	EU021615	EU982048	EU021674	EU021653	NT	NT
*A. awamori *	FRR 4795 = ATHUM 5181	Unrecorded source	Unknown	EU982010	EU982035	EU982069	EU982094	EU982117	EU982150
*A*. *bisporus* ^T^	NRRL 3693	Soil stored 10 years at 30°C	USA	EF661208	EU982052	EF661121	EF661077	EU982133	EU982166
*A. brunneouniseriatus *	NRRL 4273	soil under *Dalbergia sissoo *	USA	EF652141	EU982054	EF652123	EF652089	EU982135	EU982168
*A*. *campestris* ^T^	NRRL 13001	Native mixed prairie soil	USA	EU014091	EU982049	EF669577	EF669619	EU982130	EU982163
*A*. *clavatoflavus* ^T^	NRRL 5113	Forest soil	Australia	EF669713	EU982058	EF669686	EF669668	NT	EU982172
*A. clavatus *	ATHUM 5032	Dairy product	Greece	EU982014	EU982039	EU982073	EU982098	EU982121	EU982154
	ATHUM 5036	Dairy product	Greece	EU982015	EU982040	EU982074	EU982099	EU982122	EU982155
*A*. *elongatus* ^T^	NRRL 5176	Alkaline soil	India	EF652502	EU982059	EF652326	EF652238	EU982139	EU982173
*A. flavus *	ATHUM 5015	Indoor air	Greece	EU982011	EU982036	EU982070	EU982095	EU982118	EU982151
	ATHUM 5033	Dairy product	Greece	EU982012	EU982037	EU982071	EU982096	EU982119	EU982152
*A. fumigatus *	ATHUM 5013	Indoor air	Greece	EU982013	EU982038	EU982072	EU982097	EU982120	EU982153
*A*. *giganteus* ^T^	NRRL 10	Bat dung	Mexico	EF669928	NT	EF669789	EF669716	EU982146	EU982181
*A*. *janus* ^T^	NRRL 1787	Soil	Panama	EU021598	EU982061	EU014076	EF669620	EU982141	EU982175
*A. niger *	ATHUM 2539	Outdoor air	Greece	EU982009	EU982034	EU982068	EU982092	EU982116	EU982149
	ATHUM 5044	Dairy product	Greece	EU982008	EU982033	EU982067	EU982093	EU982115	EU982148
*A. niveus *	ATHUM 5029	Indoor air	Greece	EU982023	EU982050	EU982082	EU982105	EU982131	EU982164
*A. ochraceus *	ATHUM 5014	Indoor air	Greece	EU982029	EU982063	EU982088	EU982111	EU982143	EU982177
*A. oryzae *	ATHUM 4958	Outdoor air	Greece	EU982022	EU982047	EU982081	EU982104	EU982129	EU982162
*A. parasiticus *	ATHUM 5037	Dairy product	Greece	EU982020	EU982045	EU982079	NT	EU982127	EU982160
	ATHUM 5038	Dairy product	Greece	EU982021	EU982046	EU982080	NT	EU982128	EU982161
*A. puniceus *	ATHUM 5434	Dairy product	Greece	EU982019	EU982044	EU982078	EU982101	EU982126	EU982159
*A*. *restrictus* ^T^	NRRL 154	Clinical isolate from cloth	England	EF652042	EU982053	EF651880	EF651978	EU982134	EU982167
*A*. *sclerotiorum* ^T^	NRRL 415	Food, fruit (apple)	USA	EF661400	EU982064	EF661337	EF661287	NT	EU982178
*A. sydowii *	ATHUM 5093	Dairy product	Greece	EU982025	EU982055	NT	NT	EU982136	EU982169
*A. terreus *	ATHUM 4761	Outdoor air	Greece	EU982024	EU982051	EU982083	EU982106	EU982132	EU982165
*A. ustus *	ATHUM 5097	Indoor air	Greece	EU982026	EU982056	EU982085	EU982108	EU982137	EU982170
	ATHUM 5103	Dairy product	Greece	EU982027	EU982057	EU982086	EU982109	EU982138	EU982171
*A. versicolor *	ATHUM 2541	Culture contaminant	Greece	EU982032	NT	EU982091	EU982114	EU982147	EU982182
*Emericella nidulans *	ATHUM 5022	Indoor air	Greece	EU982031	EU982066	EU982090	EU982113	EU982145	EU982180
*E. variecolor *	ATHUM 5019	Indoor air	Greece	EU982030	EU982065	EU982089	EU982112	EU982144	EU982179
*Eurotium amstelodami *	ATHUM 5082	Indoor air	Greece	EU982017	EU982042	EU982076	NT	EU982124	EU982157
*E. rubrum *	FRR 0326 = ATHUM 5183	Stored pack of prunes	Australia	EU982018	EU982043	EU982077	NT	EU982125	EU982158
*Neosartorya fischeri *	ATHUM 5030	Dairy product	Greece	EU982016	EU982041	EU982075	EU982100	EU982123	EU982156
*Metarhizium anisopliae *				AF218207	AF218207	AY995134	DQ522453	AY884128	AY884128

^a^ATHUM: Culture Collection of Fungi, National and Kapodistrian University of Athens.

^b^NRRL: National Center For Agricultural Utilization Research.

NT: nontested.

Type strains designated by a superscript T.

**Table 2:** Phenotypic characters of* Aspergillus *species originating from Greece.

Species	ATHUM number	Vesicle shape	Vesicles (*μ*m)	Metulae (*μ*m)	Phialides (*μ*m)	Conidia	
						Size (*μ*m)	Ornamentation
*Aspergillus aculeatus *	5028	Globose	36–50	—	6–10 × 3.5–5	4-5	Finely rough
*Aspergillus clavatus *	5032, 5036	Clavate	15–150 × 15–55	—	7–10 × 2.5–4	4-5(-8) × 2.8–4	Smooth
*Aspergillus flavus *	5015	Globose	30–55	9–13 × 4-5	9–14 × 2-3	3–6	Smooth
*Aspergillus fumigatus *	5013	Spathulate	(13-)17–22	—	6–9 × 2-3	2.2–3.5	Minutely echinulate
*Aspergillus niger *	2539, 5044	Globose	40–65	14–22 × 4–6	7–9 × 3-4	4–5.5	Rough
*Aspergillus niveus *	5029	Spathulate	9–16	6-7 × 2-3	6-7 × 2-3	2-3	Smooth
*Aspergillus ochraceus *	5014	Globose	22–40	10–15 × 4-5	10-11 × 2-3	2-3	Smooth
*Aspergillus oryzae *	4958	Subglobose	25–45	9–12 × 4-5	9–12 × 4–6	5–8	Rough
*Aspergillus parasiticus *	5037, 5038	Globose	25–40	—	7–11 × 3-4	3.5–6	Rough
*Aspergillus sydowii *	5093	Pyriform	8–17	4–6(-7) × 3-4	6–8 × 2-3	3-4	Echinulate
*Aspergillus terreus *	4761	Globose	10–17	5-6 × 2-3	7-8 × 1.5–2	2–2.5	Smooth
*Aspergillus ustus *	5097, 5103	Pyriform	11–17	5–7 × 3.5–4	4–7 × 2.5–3.5	3-4	Rough
*Aspergillus versicolor *	2541	Pyriform	10–15 × 3–5	5–7 × 3–5	5–8 × 3–5	3-4	Rough
*Emericella nidulans *	5022	Spathulate	10–14	7-8 × 3-4	6-7 × 2-3	3-4	Rough
*Emericella variecolor *	5019	Spathulate	16–19	6–9 × 2-3	6–9 × 2-3	2-3	Rough
*Eurotium amstelodami *	5082	Subglobose	15–35	—	6–9 × 3-4	4–6	Echinulate
*Neosartorya fischeri *	5030	Spathulate	12–16	—	6-7 × 2–2.5	2-3	Smooth
