# Supplementary figures and images for: A Phylogenetic Analysis of Greek Isolates of Aspergillus Species Based on Morphology and Nuclear and Mitochondrial Gene Sequences
Source: Biomed Res Int. 2013 May 9;2013:260395. doi: 10.1155/2013/260395 (PMC3665174; doi:10.1155/2013/260395)

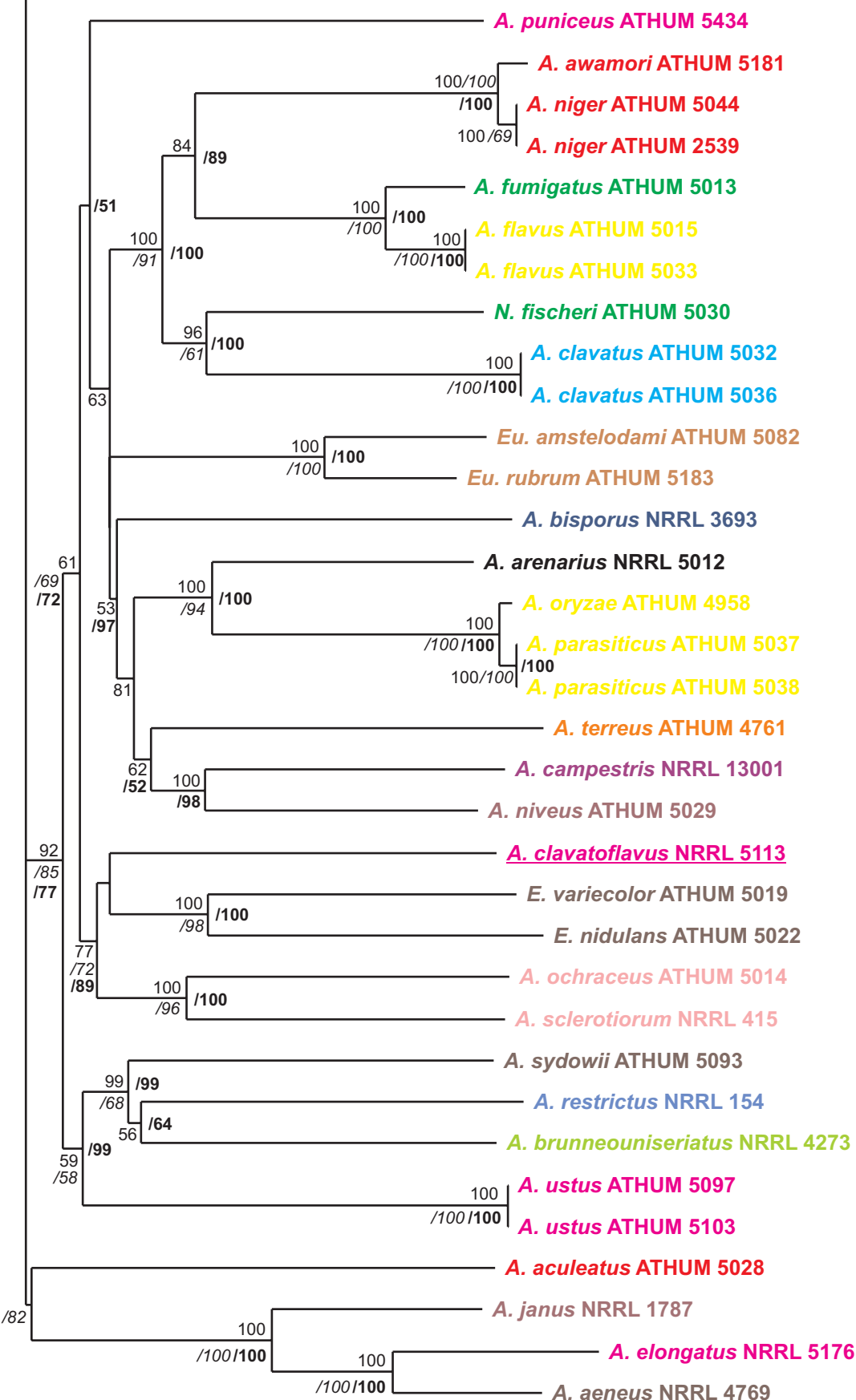

0.1

Fig S1

Supplement: Supplementary file 1 — Supplemental Figure 1: Phylogenetic tree constructed from unambiguously aligned DNA sequences of the nuclear IGS region as produced by NJ. Sequences obtained during this study are presented in bold, whereas those retrieved from GenBank are shown in roman with their Accession Numbers. Clade credibility using NJ calculated from 1,000 replicates (numbers in roman), parsimonial BS support calculated from 100 replicates (numbers in italics) using PAUP and PPs produced by 1,000,000 generations (numbers in bold) using MrBayes, are shown. Supplemental Figure 2: Phylogenetic tree constructed from unambiguously aligned DNA sequences of the mitochondrial cox1 gene as produced by NJ. Sequences obtained during this study are presented in bold, whereas those retrieved from GenBank are shown in roman with their Accession Numbers. Clade credibility using NJ calculated from 1,000 replicates (numbers in roman), parsimonial BS support calculated from 100 replicates (numbers in italics) using PAUP and PPs produced by 1,000,000 generations (numbers in bold) using MrBayes, are shown. [file 260395.f1.pdf]

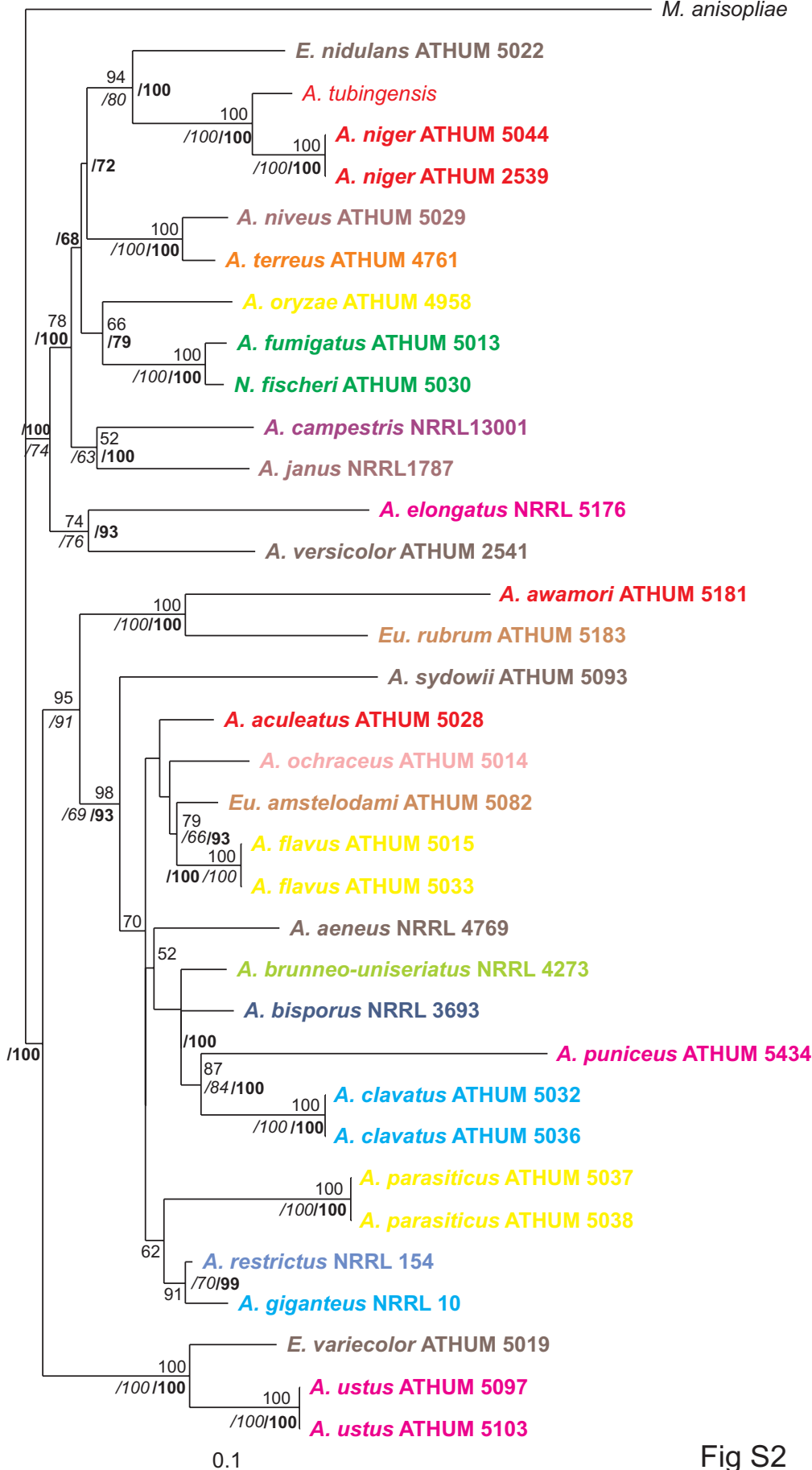

Fig S2

Supplement: Supplementary file 2 [file 260395.f2.pdf]
